# Association Between the Use of Pre- and Post-thrombolysis Anticoagulation With All-Cause Mortality and Major Bleeding in Patients With Pulmonary Embolism

**DOI:** 10.3389/fcvm.2022.880189

**Published:** 2022-06-30

**Authors:** Jiang-Shan Tan, Ningning Liu, Song Hu, Yan Wu, Xin Gao, Ting-Ting Guo, Xin-Xin Yan, Fu-Hua Peng, Lu Hua

**Affiliations:** ^1^Center for Respiratory and Pulmonary Vascular Diseases, Department of Cardiology, Key Laboratory of Pulmonary Vascular Medicine, National Clinical Research Center of Cardiovascular Diseases, State Key Laboratory of Cardiovascular Disease, Fuwai Hospital, National Center for Cardiovascular Diseases, Chinese Academy of Medical Sciences and Peking Union Medical College, Beijing, China; ^2^Peking University Sixth Hospital/Institute of Mental Health, Beijing, China; ^3^National Health Council Key Laboratory of Mental Health (Peking University), National Clinical Research Center for Mental Disorders, Peking University Sixth Hospital, Beijing, China

**Keywords:** anticoagulation (AC), thrombolysis/thrombolytic agents, all-cause mortality, major bleeding, pulmonary embolism

## Abstract

**Objective:**

To explore the comparative clinical efficacy and safety outcomes of anticoagulation before (pre-) or following (post-) thrombolytic therapy in systemic thrombolytic therapy for pulmonary embolism (PE).

**Methods:**

PubMed, the Cochrane Library, EMBASE, EBSCO, Web of Science, and CINAHL databases were searched from inception through 1 May 2021. All randomized clinical trials comparing systemic thrombolytic therapy vs. anticoagulation alone in patients with PE and those that were written in English were eligible. The primary efficacy and safety outcomes were all-cause mortality and major bleeding, respectively. Odds ratios (OR) estimates and associated 95% confidence intervals (CIs) were calculated. A Bayesian network analysis was performed using R studio software, and then the efficacy and safety rankings were derived.

**Results:**

This network meta-analysis enrolled 15 trials randomizing 2,076 patients. According to the plot rankings, the anticoagulant therapy was the best in terms of major bleeding, and the post-thrombolysis anticoagulation was the best in terms of all-cause mortality. Taking major bleeding and all-cause mortality into consideration, the most safe–effective treatment was the post-thrombolysis anticoagulation in patients who needed thrombolytic therapy. The net clinical benefit analysis comparing associated ICH benefits vs. mortality risks of post-thrombolysis anticoagulation demonstrated a net clinical benefit of 1.74%.

**Conclusion:**

The systemic thrombolysis followed by anticoagulation had a better advantage in all-cause mortality and major bleeding than the systemic thrombolysis before anticoagulation. The adjuvant anticoagulation treatment of systemic thrombolytic therapy should be optimized.

## Introduction

Pulmonary embolism (PE) commonly occurs in the general community, often resulting in high morbidity and mortality (1–3). Systemic thrombolytic therapy has become an established procedure (4), which can recirculate occluded pulmonary arteries, salvage pulmonary circulation, and reduce mortality. However, a high level of vigilance is needed due to the high frequency of major bleeding complications in patients with systemic thrombolytic treatment. Therefore, the role of systemic thrombolytic therapy remains controversial in non-high-risk/fatal PE. Bleeding can be not only induced by the thrombolytic agent itself but also results from adjunctive therapy with anticoagulation or other risk factors, such as advanced age and hypertension (5). Efforts have been made to adjust the thrombolytic agent or thrombolytic approach to reduce the risk of major bleeding associated with systemic thrombolytic therapy, such as reducing the dose of thrombolytic drugs (6, 7) or catheter-directed thrombolysis (8).

The dynamic balance between thrombosis and thrombolysis is influenced by both optimization of the thrombolysis and the adjunctive antithrombotic therapy. There are two administrations of anticoagulation agents including unfractionated heparin (UFH) or low-molecular-weight heparin (LMWH), which can be started before thrombolysis (pre-thrombolysis anticoagulation) and continuing (9) or started after thrombolysis (post-thrombolysis anticoagulation) according activated partial thromboplastin time (aPTT) (10). The aggressive adjunctive therapy with heparin has been identified as that which increases the risk of major bleeding associated with thrombolytic therapy (11). However, we neglected the effect of the sequence between anticoagulation and thrombolytic therapy on major bleeding. Several randomized, controlled trials have compared the safety and efficiency between heparin and thrombolytic agents in patients with an acute PE (1), but a beneficial effect of pre- and post-thrombolysis anticoagulation on important clinical outcomes is difficult to demonstrate. Therefore, the efficacy and safety of these two anticoagulation strategies of systemic thrombolytic therapy are unclear in patients with acute PE.

To determine whether the treatment effect of thrombolysis with different adjunctive anticoagulation truly exists, we performed this network analysis in the hope of obtaining the optimized anticoagulant therapy of systemic thrombolysis by pooling the results of the available randomized, controlled trials.

## Methods

Search strategy, study selection, data extraction, and analysis of our study were all performed based on a pre-defined protocol (Supplementary Material 1).

### Search Strategy

**FIGURE 1:**
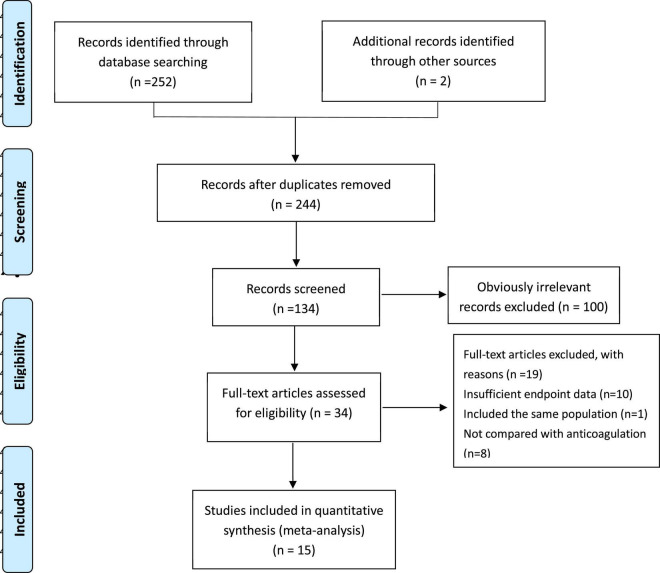
Search strategy and study selection.

PRISMA (Preferred Reporting Items for Systematic reviews and Meta-Analyses) statement (12) was referred for a systematic literature review. Two authors (J.S. Tan and N.N. Liu) systematically performed an electronic literature search in PubMed, the Cochrane Library, EMBASE, EBSCO, Web of Science, and CINAHL databases (reported the outcomes within 30 days or in hospital, written in English and published from inception through 1 May 2021; Supplementary Methods 1). All the randomized controlled trials were included, which compared a thrombolytic agent [desmoteplase, recombinant tissue plasminogen activator (alteplase), reteplase, streptokinase, tenecteplase, or urokinase] administered systemically by the i.v. route and heparin (low-molecular-weight heparin, unfractionated, fondaparinux, or vitamin K antagonist) with heparin alone in patients with PE. To get a literature search as comprehensive as possible, reference lists from retrieved articles and reference literature (including systematic reviews and guidelines) were examined (Figure 1).

### Study Selection and Data Extraction

All randomized controlled trials comparing thrombolytic therapy with anticoagulation alone (Supplementary Methods 2) in patients with PE were included. We excluded the studies using mechanical thrombectomy along with local catheter-delivered thrombolysis or thrombolytic treatment or those just comparing two regimens of thrombolytic therapy. The possible trials were independently evaluated by two authors (J.S. Tan and N.N. Liu). We excluded the non-relevant studies by screening the title and abstract. The full text was independently screened by two authors (J.S. Tan and N.N. Liu) to assess the study eligibility and they extracted related data (study design and patient characteristics) according to the pre-designed protocol. Once a disagreement about study inclusion or data extraction occurred, it would be resolved by consensus or by a discussion with another author (Dr. Hua).

### Outcomes and Measurements

All the outcomes that occurred within 30 days or in the hospital were recorded in the present study. The primary efficacy and safety outcomes were all-cause mortality and major bleeding events, respectively. The secondary safety outcomes were intracranial hemorrhage (ICH). Recurrent PE (confirmed by a validated diagnostic examination) and composite outcomes (including major bleeding, recurrent PE, and all-cause mortality; Supplementary Methods 3) were considered the secondary efficacy outcomes.

The definition of major bleeding refers to the International Society of Thrombosis and Hemostasis (ISTH) if sufficient values were available. In other cases, major bleeding was defined according to the original studies. Both trial and patient characteristics, and outcomes were independently extracted from included studies by two authors (J.S. Tan and N.N. Liu).

**FIGURE 2:**
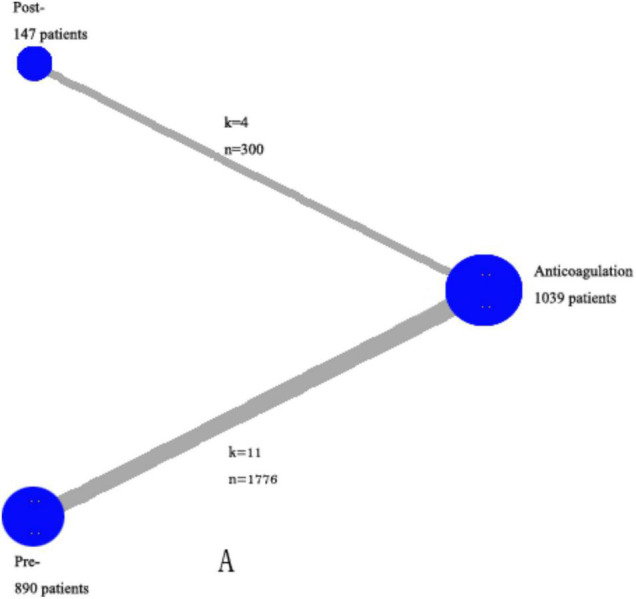
Network of the comparisons for the Bayesian network analysis. The size of the nodes is proportional to the number of patients (in parentheses) randomized to receive the treatment. The width of the lines is proportional to the number of trials (beside the line) comparing the connected treatments. However, we excluded the trials in which both events in the experimental and control groups were 0 in specific analysis. k—number of trials per comparison; n—number of patients per comparison.

As is shown in Figure 2, all-cause mortality was evaluated in 15 studies (7, 9, 10, 13–24) that satisfy the inclusion criteria. Reporting of ICH, major bleeding events, recurrence, and comprised outcomes were completed by variable studies, and not every study presented all data.

### Study Quality and Risk of Bias Assessment

According to the Cochrane Handbook of Systematic Reviews (25), study quality and the risk of bias were evaluated and they specifically concentrated on the following criteria: (1) proper sequence generation, (2) proper allocation concealment, (3) blinding of the investigator assessing clinical outcomes and the patients, (4) proper outcomes assessment, and (5) short time clinical events recorded during the hospitalization or within 1 month.

### Statistical Analysis

Data for further statistical analysis was the intention to treat. The model-used (fixed vs. random-effects) was determined according to the lowest deviance information criterion (DIC) for individual outcomes. Odds ratios (OR) estimates and associated 95% confidence intervals (CIs) were calculated for meta-analysis. We excluded the studies which have 0 events in both arms because they do not contribute to the overall effect. The Bayesian network meta-analysis was performed using R studio software, and the effective and safe treatment rankings were derived.

### Sensitivity Analysis

The included trials have been strictly screened by the including criteria. Sensitivity analysis did not repeat for outcomes.

### Statistical Heterogeneity and Convergence Assessment

Visual inspection of the forest plots was used to investigate the possibility of statistical heterogeneity, and the *I*^2^ was used to measure heterogeneity (26) [*I*^2^ < 25% was considered mild, *I*^2^ < 75% was moderate, and *I*^2^ > 75% was severe (26)]. Brooks–Gelman–Rubin diagnosis plot and Trace plot were used to diagnose the convergence of the model. Ranking histograms were used to show the ranking possibility for each anticoagulation strategy. In this analysis, a 2-sided *P* < 0.05 was statistically significant. All analyses were performed using R i386 (version 3.2.2, 3 chains were used, including 1,50,000 burn-in iterations followed by 2,00,000 iterations) and SPSS V 24.0 (SPSS Statistics v. 24.0, SPSS Inc.) (Supplementary Methods 4).

### Net Clinical Benefit

Besides, a net clinical benefit analysis was performed for choosing pre- or post-thrombolysis anticoagulation in systemic thrombolytic therapy for PE. We calculated the short-term risk of ICH (Ti) prevented by post-thrombolysis anticoagulation minus the short-term mortality (Tm) induced by post-thrombolysis anticoagulation. Then, the former was multiplied by a weighting factor of 0.75, suggesting that a single ICH event amounted to 75% of the effect of single mortality. The weighting factor was referred to the related data which demonstrated the serious disability or probability of death owing to ICH (27). The weighting factor was used to provide an accurately and comprehensively conservative estimate of potential benefits associated with post-thrombolysis anticoagulation. The following equation illustrates this definition: net clinical benefit = weighing factor × (Ti_pre–_–Ti_post–_) – (Tm_post–_–Tm_pre–_) (1).

## Results

### Study Search and Study Characteristics

Overall, 367 records were identified through database searching and 34 were considered eligible through title and abstract with 15 Randomized controlled trials (RCTs) in the final meta analysis.

As is shown in Figure 2, all-cause mortality was evaluated in 15 studies (7, 9, 10, 13–24) that met our inclusion criteria. The detailed selection process is shown in Figure 1. Totally, 2,076 patients were enrolled in our analysis. Eleven trials were defined as the pre-thrombolysis anticoagulation group, anticoagulation before thrombolytic therapy in PE. The other trials, anticoagulation following thrombolytic therapy, were defined as post- group (Figure 2). The baseline characteristics for every single trial are shown in Table 1.

**TABLE 1:** Baseline characteristics of trials.

Source	No. of patients	Age, mean (Range or SD)	Male, No. (%)	Type of PE	Thrombolysis	Comparator	Pre- or Post-anticoagulation	Major bleeding criteria	Follow-up^1^ (d)	Outcomes^3^
A Cooperative Study (38)	160	45.0 (<50), 55.0 (>50)	92 (57.3)	All	Urokinase (2,000 U/lb, then 2,000 U/lb/h for 12 h)	Heparin	Post-	Hematocrit	14	ECH, Major, All-cause Mortality, Recurrence, Comprised outcome
Ly et al. (24)	25	53.2 (23–70)	11 (44.0)	All	Streptokinase 72 h	Heparin	Pre-	Not pre-specified	10	ECH, Major, All-cause Mortality, Recurrence, Comprised outcome
Becattini et al. (15)	58	68.2 (4.3)	23 (39.7)	Stable	Tenecteplase (30–50 mg) plus heparin	Heparin	Pre-	Bleeding need transfusion, surgical control or fatal or ICH	30	ICH, ECH, Major, All-cause Mortality, Recurrence, Comprised outcome
Dotter et al. (23)	31	18–85^a^	12 (38.7)	All	Streptokinase (250,000 IU in 5% dextrose/20–30 min, followed 100,000 IU/hour for 18–72 h)	Heparin	Post-	Not pre-specified	DH^2^	ECH, Major, All-cause Mortality, Recurrence, Comprised outcome
Dalla-Volta et al. (18)	36	64.7 (12.5)	12 (33.3)	Stable	Alteplase (100 mg/2 h) plus	Heparin	Pre-	ICH or ≥ 1(units PRBCs transfusion	30	ICH, ECH, Major, All-cause Mortality, Recurrence, Comprised outcome
Fasullo et al. (16)	72	56.0 (16.1)	41 (56.9)	Stable	Alteplase (100 mg)	Heparin	Pre-	Bleeding need transfusion, surgical control or fatal or ICH	10	ECH, Major, All-cause Mortality, Recurrence, Comprised outcome
Goldhaber et al. (10)	101	58.5 (16.9)	44 (44)	Stable	Alteplse (100 mg)		Post-	ICH, need for surgery	14	ICH, ECH, Major, All-cause Mortality, Recurrence, Comprised outcome
Jerjes-Sanchez et al. (13)	8	51.0 (22.9)	5 (63)	All	Streptokinase (1,500,000 IU)	Heparin	Post-	Not pre-specified	30	All-cause Mortality, Comprised outcome
Kline et al. (17)	83	55.4 (14.0)	49 (59.0)	Stable	Tenecteplase (30–50 mg/2 h)	LMWH	Pre-	Not pre-specified	5	ICH, ECH, Major, All-cause Mortality, Recurrence, Comprised outcome
Konstantinides et al. (19)	256	62.1 (10.5)	122 (47.6)	Stable	Alteplase (100 mg/2 h)	Heparin	Pre-	Fatal, hemorrhagic stroke, hemoglobin drop ≥ 4 g per deciliter.	DH	ECH, Major, All-cause Mortality, Recurrence, Comprised outcome
Levine et al. (20)	58	60.7 (3.2)	29 (50.0)	Stable	Alteplase (0.6 mg/kg/2 min of ideal body weight)	Heparin	Pre-	Hemoglobin drop > 20 g/L [ ≥ 2(units PRBCs, retroperitoneal or ICH)]	10	All-cause Mortality, Comprised outcome
Meyer et al. (9)	1,005	66.2 (15.3)	473 (47.1)	Stable	Tenecteplase (30–50 mg)	Heparin	Pre-	Bleeding need transfusion, surgical control and fluid replacement or fatal.	7	ICH, ECH, Major, All-cause Mortality, Recurrence, Comprised outcome
PIOPED Investigators (21)	13	59.3 (16.2)	9 (75.0)	Stable	Alteplase (40–80 mg)	Heparin	Pre-	Not pre-specified	30	ECH, Major, All-cause Mortality
Sharifi et al. (7)	121	58.5 (9.5)	55 (45.5)	Stable	Alteplase (50 mg/2 h)	Heparin or LMWH	Pre-	Not pre-specified	DH	All-cause Mortality, Comprised outcome
Taherkhani et al. (14)	50	55.7 (12.4)	20 (40.0)	Stable	Alteplase (100 mg/90 min) or Streptokinase (1,500,000 u/2 h)	Enoxaparin	Pre-	Fatal, hemorrhagic stroke, hemoglobin drop ≥ 4 g per deciliter	DH	All-cause Mortality, Comprised outcome

*^1^The follow-up days did not mean the whole follow-up time in the articles, it just meant the shortest recording events’ time in their articles. It often means during the hospital stay or the recorded events’ time, which is no more than 1 month.*

*^2^“DH” means the follow-up was finished during hospitalization.*

*^3^Outcomes means those events of this trial were counted in the calculation. ICH, intracranial hemorrhage; ECH, extracranial hemorrhage; Mod, intermediate risk (hemodynamically stable with objective evidence of right ventricular dysfunction). ^a^Only age range is available but without mean age.*

### Risk of Bias and Publication Bias

Of the 15 included trials, 7 (46.67%) were assessed as low risk of bias in all the domains. Two (13.33%) were at a high risk of bias for blinding and one (6.67%) for allocation concealment. No studies were at high risk of bias for sequence generation, detection bias, and attrition (Supplementary Figure 1 and Supplementary Table 2). No evidence was proved of publication bias (Supplementary Figure 2).

### Primary Efficiency Outcome: All-Cause Mortality

**FIGURE 3:**
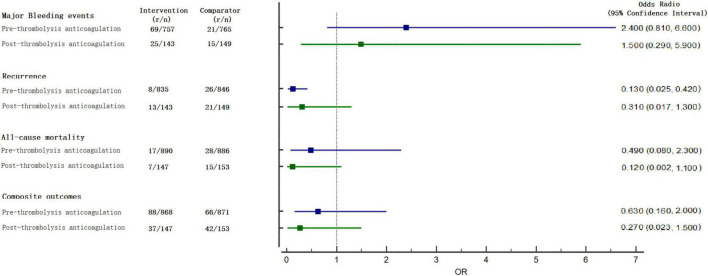
Forest plots for relative effect as compared with anticoagulation.

For all-cause mortality, 15 studies reported at least 1 event in any group and 2,076 enrolled patients. There were 64 deaths: 17 (1.91%) of 890 patients in the pre-group, 7 (4.76%) of 147 patients in the post- group, and 43 (4.14%) of 1,039 in the anticoagulation group. Compared with the anticoagulation group, both pre- group [OR, 0.490 (0.080, 2.300)] and post- group [OR, 0.120 (0.002, 1.100)] were not associated a difference in all-cause mortality (Figure 3).

### Primary Safety Outcome: Major Bleeding Events

For major bleeding events, 11 studies reported at least 1 event in any group and 1,814 enrolled patients. There were 130 major bleeding events: 69 (9.11%) of 757 patients in the pre-group, 25 (17.48%) of 143 patients in the post- group, and 36 (3.94%) of 914 in the anticoagulation group. Both pre- [OR, 2.400 (0.810, 6.600)] and post-group [OR, 1.500 (0.290, 5.900)] were not associated with a significant difference (Figure 3) when compared with anticoagulation alone.

### Secondary Outcomes

**FIGURE 4:**
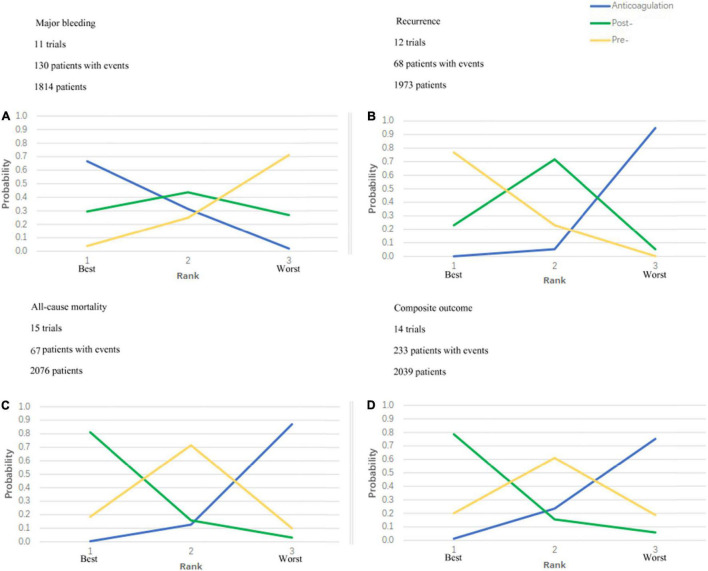
Ranking plots. Strategy ranking plots for primary and secondary outcomes are stratified by treatment. **(A)** Is the ranking plot for major bleeding; **(B)** is the plot for recurrence; **(C)** is the plot for all-cause mortality and **(D)** is the plot for composite outcome. Each line represents 1 strategy and shows the probability of its ranking from best to worst. The peak of the line represents the rank that the strategy is most likely to be for each given outcome. For example, for all-cause mortality, post- thrombolytic anticoagulation is most likely to rank best; pre- thrombolytic anticoagulation, second best; and anticoagulation, worst.

Secondary outcomes were not reported in all trials. The detailed analysis results are shown in Figures 3, 4.

### Second Efficiency Outcome: Recurrence and Comprised Outcomes

For recurrence, the pre-group [OR, 0.013 (0.025, 0.420)] significantly decreased the risk, but no statistical significance was observed in the post-group [OR, 0.310 (0.017, 1.300); Figure 3] when compared with anticoagulation alone. Compared with anticoagulation alone, both pre-group [OR, 0.630 (0.160, 2.000)] and post-group [OR, 0.270 (0.023, 1.500)] were not associated with a difference in comprised outcomes (Figure 3).

### Secondary Safety Outcome: Intracranial Hemorrhage

Five studies reported at least 1 event in any group and 1,283 patients were enrolled in the ICH analysis. In all, 36 patients were with ICH: 29 (4.88%) of 594 patients in the pre-group, 0 (0%) of 46 patients in the post- group, and 7 (1.09%) of 643 in the anticoagulation group. Due to the small sample size and 0 events in the post-group, we were limited in any further statistical analysis.

### Strategy Class Rankings

**FIGURE 5:**
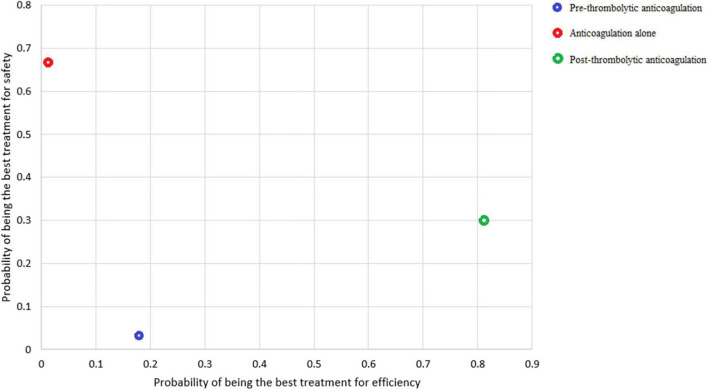
Ranking plot in consideration of efficiency and safety.

Figure 4 shows the ranking probabilities of each treatment in the 3 possible positions. As is shown in Figure 4, recommended ranking in all-cause mortality: post-thrombolysis anticoagulation > pre-thrombolysis anticoagulation > anticoagulation alone; major bleeding: anticoagulation alone > post-thrombolysis anticoagulation > pre-thrombolysis anticoagulation; recurrent PE: pre-thrombolysis anticoagulation > post-thrombolysis anticoagulation > anticoagulation alone; composite outcome: post-thrombolysis anticoagulation > pre-thrombolysis anticoagulation > anticoagulation alone. Post-thrombolysis anticoagulation was the most beneficial treatment just in consideration of all-cause death (0.81) and combined end-point events (0.79). Based on the ranks of effectiveness and safety, the post-thrombolysis anticoagulation was the best in terms of both major bleeding and all-cause mortality (Figure 5).

### Heterogeneity and Convergence Assessment

Brooks-Gelman-Rubin diagnosis plot (Supplementary Figure 4) and Trace plot (Supplementary Figure 5) showed that the convergence of the model was good. Heterogeneity test results showed that heterogeneity was low or acceptable, except for combined outcomes.

### Net Clinical Benefits

The net clinical benefit analysis comparing associated ICH benefits vs. mortality risks of post-thrombolysis anticoagulation demonstrated a net clinical benefit of 0.0174 (0.0001, 0.0365). This means the net clinical benefit analysis comparing associated ICH benefits vs. mortality risks of post-thrombolysis anticoagulation demonstrated a net clinical benefit of 17.4%_0_.

## Discussion

To our knowledge, there are no RCTs focused on this topic up to now, and this is the first study to explore the efficiency and safety of systemic thrombolysis with pre-thrombolysis anticoagulation or post-thrombolysis in unselected patients with acute PE. In the recommended ranking, systemic thrombolysis followed by anticoagulation was the most beneficial treatment in consideration of all-cause death and combined end-point events, demonstrating a net clinical benefit of 17 fewer deaths per 1,000 people when compared with systemic thrombolysis before anticoagulation.

Major bleeding is an important and apprehensive conundrum for clinicians when choosing thrombolytic therapy in patients with PE. Several meta-analyses have assessed the risk of major bleeding associated with thrombolysis in patients with PE (1, 28, 29). Thabut et al. showed that thrombolytic therapy did lead to a near doubling in the rate of major hemorrhage with a significant reduction in mortality or the recurrence of PE as compared with heparin when administered to unselected patients with acute PE (30). Chatterjee et al. also showed that thrombolytic therapy was associated with lower rates of all-cause mortality but increased risks of major bleeding and ICH among patients with PE (1). Thrombolytic therapy may help reduce mortality but may cause major hemorrhagic events and stroke (31). It should be pointed out that all these previous meta-analyses included the clinical trials of systemic thrombolysis with pre-thrombolysis and post-thrombolysis anticoagulation.

The thrombolytic therapy of PE has followed a similar path to that of myocardial infarction (MI), including adjunctive anticoagulation therapy (9). Heparin should not be infused concurrently with streptokinase or urokinase. For tPA or reteplase, concurrent use of heparin is optional (32). In clinical practice, systemic thrombolysis with pre-thrombolysis anticoagulation was the favored thrombolytics treatment. Eleven clinical trials of the total fifteen trials of our study selected pre-thrombolysis anticoagulation while only four trials selected post-thrombolysis anticoagulation. But the hemorrhagic complications of thrombolytic therapy were higher in PE than that in MI (11). One hypothesis to explain the higher rate of hemorrhagic complications following thrombolytic therapy in patients with PE was that venous congestion and an increase in central venous pressure could increase the bleeding risk when PE induces acute cor pulmonale with hemodynamic compromise (33). However, this is just a hypothesis, and there is no strong evidence to support it. Besides, in a patient with ST-elevation infarction, due to the use of heparin, antiplatelet agents, and thrombolytic therapy, the trend of physicians is to avoid punctures in major veins. However, this will not happen in PE where patients are taken for punctures to place a central line and for arterial blood gases, which sometimes includes punctures in the femoral arteries. Therefore, the involvement of arterial and venous punctures may be another possible mechanism of hemorrhagic complications. Furthermore, the well-known risk factor for hemorrhagic complications, liver dysfunction, which induces clotting disorders (34) caused by liver injury due to a combination of arterial hypoxemia, low cardiac output, and liver congestion could be a major factor for the risk of bleeding in patients with acute cor pulmonale and circulatory failure (35). Therefore, we should reexamine the adjunctive anticoagulation therapy of systemic thrombolysis in PE to decrease bleeding events.

We have made the first try to analyze whether pre- and post-thrombolysis anticoagulation could make a difference for patients with PE. Our results revealed that the systemic thrombolysis with post-thrombolysis anticoagulation reduced both all-cause mortality and combined endpoints (Figure 4) when compared with anticoagulation alone and systemic thrombolysis with pre-thrombolysis anticoagulation in the ranking plots. Although systemic thrombolysis with post-thrombolysis anticoagulation increased the risk of major bleeding when compared with anticoagulation alone, it is noteworthy that post-thrombolysis anticoagulation reduced the risk when compared with pre-thrombolysis anticoagulation. The international PEITHO (Pulmonary Embolism Thrombolysis) trial (9) enrolled 1,006 patients (506 patients in the tenecteplase group and 499 in the placebo group) with confirmed PE and concluded that in patients with intermediate-risk PE, fibrinolytic therapy could reduce the risk of hemodynamic decompensation, but great caution should be warranted given an increased risk of major hemorrhage and stroke. However, it is worth noting that the anticoagulant administration was started immediately after randomization (also referred to as pre-thrombolysis anticoagulation in our study) in the PEITHO study. In the present meta-analysis, ICH occurred in 29 (4.88%) of the 594 patients in the pre-thrombolysis anticoagulation group, but none occurred in the post-thrombolysis anticoagulation group. In combination with the ranking plots of major bleeding and all-cause mortality, post-thrombolysis anticoagulation seems more favorable than pre-thrombolysis anticoagulation.

In brief, two anticoagulation strategies had differences in safety and effectiveness. If the results of our meta-analysis are confirmed by future randomized clinical trials, there may be a shift in the adjuvant anticoagulation treatment of patients with PE using thrombolytics. Besides, it is also a challenge for researchers to explore other concomitant anticoagulants with thrombolytics, such as the “direct oral anticoagulants (DOAC),” in hemodynamically stable PE (36). Furthermore, previous studies have revealed that fibrinolytic therapy (FT) in patients with PE could accelerate the reversal of right ventricular dysfunction if the patients were properly selected (10) and the weight-adjusted unfractionated heparin regimen was also regarded as a strategy to reduce bleeding complications (37). Future research should concentrate on the probability to accrue maximal clinical benefits by minimizing the risk of bleeding for intermediate-risk PE.

Our study has several limitations which must be taken into consideration for accurate interpretation of the reported efficiency and safety. Firstly, there are no RCTs to compare efficiency and safety between pre- and post-thrombolysis anticoagulation, which means there may be an unequal distribution of potentially confusing factors, and most importantly, the potential imbalance of risk for bleeding between pre- and post-thrombolysis anticoagulation groups. Although the characteristics of the enrolled patients were seemingly matched with each other, the matching degree of basic data was not strict and accurate as RCTs. However, our data were all collected from the RCTs which concentrated on anticoagulants in conjunction with thrombolytics. There were similarly explicit inclusion and exclusion criteria in different RCTs. Secondly, the bias in sample size among different groups included in the present study exists, and the sample size of post-thrombolysis anticoagulation is small than the other two groups. Thirdly, the anticoagulants (heparin or low molecular weight heparin) and thrombolytic agent (such as urokinase, streptokinase, or rtPA) included in the study were inconsistent. Strict criteria for study selection and proper management for pooled data according to QUORUM guidelines and recognized recommendations were employed to emphasize this issue, and the heterogeneity was tested by summary.anohe plot. The heterogeneity was low or acceptable, except for the *I*^2^ in combined end-point events. The presumptive reason is that the combined end-point event was a collection of heterogeneities, though the heterogeneity in combined end-point events would be very high. Thus, bias is unlikely to occur in patient selection and publication. Fourthly, no solicitude was shown for differences in study quality, as all included studies were considered as moderate to good methodological quality. Lastly, the protocol was not prospectively registered in PROSPERO.

## Conclusion

The systemic thrombolysis following anticoagulation had a better advantage in all-cause mortality and major bleeding than the systemic thrombolysis before anticoagulation. Therefore, this meta-analysis suggested that early institution of thrombolysis, whenever indicated (without waiting and hesitating for long periods giving anticoagulation alone), maybe a safer approach to reduce the all-cause mortality and major bleeding. However, this study is hypothesis-generating, and a controlled study is required to know the true participation of pre- and post-thrombolysis anticoagulation in the incidence of hemorrhagic complications.

## Data Availability Statement

The original contributions presented in this study are included in the article/Supplementary Material, further inquiries can be directed to the corresponding author/s.

## Author Contributions

LH and J-ST: full access to all of the data in the study and took responsibility for the integrity of the data and the accuracy of the data analysis, and study concept and design. J-ST, NL, YW, XG, T-TG, X-XY, F-HP, and SH: drafting of the manuscript. J-ST, NL, YW, and XG: critical revision of the manuscript for important intellectual content. J-ST and NL: statistical analysis. LH, NL, X-XY, F-HP, and SH: administrative, technical, or material support. LH: study supervision. All authors contributed to the article and approved the submitted version.

## Conflict of Interest

The authors declare that the research was conducted in the absence of any commercial or financial relationships that could be construed as a potential conflict of interest.

## Publisher’s Note

All claims expressed in this article are solely those of the authors and do not necessarily represent those of their affiliated organizations, or those of the publisher, the editors and the reviewers. Any product that may be evaluated in this article, or claim that may be made by its manufacturer, is not guaranteed or endorsed by the publisher.
