# Supplementary material for: Association Between the Use of Pre- and Post-thrombolysis Anticoagulation With All-Cause Mortality and Major Bleeding in Patients With Pulmonary Embolism
Source: Front Cardiovasc Med. 2022 Jun 30;9:880189. doi: 10.3389/fcvm.2022.880189 (PMC9279684; doi:10.3389/fcvm.2022.880189)
Supplement: Supplementary file 1 [file Data_Sheet_1.docx]

**Meta-analysis protocol**

**Aim**

To evaluate all-cause mortality benefits, intracranial hemorrhage (ICH) and other outcomes of Post- and Pre-thrombolysis anticoagulation for pulmonary embolism using a bayesian network analysis.

**Population**

Patients with pulmonary embolism

**Intervention**

Dosed with systemic unfractionated heparin (UFH) or low-molecular-weight heparin (LMWH) before (Pre-) thrombolytic therapy

Dosed with systemic unfractionated heparin (UFH) or low-molecular-weight heparin (LMWH) following (Post-) thrombolytic therapy

**Comparison**

Control: Placebo or Anticoagulation alone

**Outcome (within 30 days or in hospital)**

Primary safety outcome

--- ICH

Primary efficacy outcome

---All-cause mortality.

The secondary safety outcome

--- Recurrent PE (confirmed by a validated diagnostic examination)

The secondary efficacy outcome

--- Composite outcome (including major bleeding, recurrent PE and all-cause mortality)

**Search strategy**

Databases searched:

- PubMed, the Cochrane Library, EMBASE, EBSCO, Web of Science, and CINAHL databases
- From database inception through Sep 30th, 2018

The initial search will be carried out by JST. And all the references will be collated on Endnote X8.

Endnote X8 should be used for removing the duplicates, then, we will finish the screening and review steps for the remaining articles:

1. Screening: Firstly, the obviously non- relevant studies should be removed by screening the title and abstract. Two authors (J.S. Tan and N.N. Liu) will finish this step without overlap, which means all of the studies will be split evenly between J.S. Tan and N.N. Liu and then they will be screened individually. Non-relevant studies should be the obviously not relevant study design, decided based on the reviewers’ discretion. If any studies will be excluded for specific reason, the authors should record them and given as “Non-relevant”. At this stage, all the reviewers should be overly inclusive so as to reduce the relevant studies omitting chances.
2. Review: After screening, all the remaining studies should be reviewed by two authors (J.S. Tan and N.N. Liu) in parallel and independently. At this stage, the principle is to assess studies strictly on the basis of inclusion and exclusion criteria. If it is necessary, we should review the full text. All the exclusion reasons should be recorded.

Additional related systematic reviews or meta should also be searched and then screened by authors for additional trials.

We provide the search terms in the eMethods 1 (Detailed Statistical Methods).

**Study inclusion criteria**

1. Randomized clinical trial
2. Comparing thrombolytic therapy with anticoagulation alone in patients with PE
3. Follow-up results within 30 days or in hospital
4. English language

Note:

Data related to the outcomes of our analyses can be used when they are from the secondary analyses of a study and the original study meets the including criteria.

If one trial is open label extension periods after the fixed randomized period, only the randomized data can be used.

**Study exclusion criteria**

1. Studies using mechanical thrombectomy along with local catheter-delivered thrombolysis or thrombolytic treatment
2. Studies just comparing two regimens of thrombolytic therapy

**Data Extraction**

Data will be extracted by two authors (J.S. Tan and N.N. Liu) in parallel and independently onto the prepared Microsoft Excel spreadsheet. Then, the data was compared within two authors to ensure the accuracy and validity of data extraction.

The following information should be extracted:

- Basic study information:
- First author, study type, publication year, journal
- Study inclusion and exclusion criteria
- All safety and efficiency outcomes:
- Event count recording in control and treatment respectively (raw numbers)

**Statistical Analysis**

Data for further statistical analysis was intention to treat. The model-used (fixed vs. random-effects) was determined according to the lowest deviance information criterion (DIC) for individual outcomes. Odds ratios (OR) estimates and associated 95% confidence intervals (CIs) were calculated for meta-analysis. We excluded the studies which have 0 events in both arms because they make no contribution to the overall effect. While, the studies with 0 events in one arm but with events in another arm, we will have a correction with 0.5 applied to the 0 events arm[^22^](#_ENREF_22). Detailed results will be presented in forest plots.

Sensitivity analysis

The included trials have been strictly screened by the including criteria. Sensitivity analysis didn’t repeat for outcomes.

Statistical heterogeneity and convergence assessment

Visual inspection of the forest plots was used to investigate the possibility of statistical heterogeneity, and the I^2^ was used to measure heterogeneity[^23^](#_ENREF_23) (I^2^ < 25% was considered mild, I^2^ < 75% was moderate and I^2^ > 75% was severe[^24^](#_ENREF_24)). Brooks-Gelman-Rubin diagnosis plot and Trace plot were used to diagnose the convergence of the model. Ranking histograms was used to show the ranking possibility for each anticoagulation strategy. In this analysis, a 2-sided P<0.05 was statistically significant. All analyses were performed using R i386 (version 3.2.2, 3 chains were used, including 150000 burn-in iterations followed by 200000 iterations), SPSS V 24.0 (SPSS Statistics v. 24.0, SPSS Inc).

Net Clinical Benefit

Besides, a net clinical benefit analysis was performed in choosing pre- or pos-thrombolysis anticoagulation in systemic thrombolytic therapy for Pulmonary Embolism. We calculated the short-term risk of ICH (Ti) prevented by pos-thrombolysis anticoagulation minus the short-term mortality (Tm) induced by pos-thrombolysis anticoagulation. Then, the former was multiplied by a weighting factor of 0.75, suggesting that a single ICH event amounted to seventy-five percent of the effect of a single mortality. The weighting factor was referred to the related data which demonstrated the serious disability or probability of death owing to ICH[^25^](#_ENREF_25). The weighting factor was used to provide an accurately and comprehensively conservative estimate of potential benefits associated with pos-thrombolysis anticoagulation. The following equation illustrates this definition: net clinical benefit = weighing factor× (Ti_pre-_−Ti_post-_)- (Tm_post-_−Tm_pre-_)[^26^](#_ENREF_26) .
