# Supplementary material for: Association Between the Use of Pre- and Post-thrombolysis Anticoagulation With All-Cause Mortality and Major Bleeding in Patients With Pulmonary Embolism
Source: Front Cardiovasc Med. 2022 Jun 30;9:880189. doi: 10.3389/fcvm.2022.880189 (PMC9279684; doi:10.3389/fcvm.2022.880189)
Supplement: Supplementary file 2 [file Data_Sheet_2.docx]

**Supplementary Online Content**

**eMethods 1. Search strategy**

**eMethods 2. Drug doses**

**eMethods 3. Event definitions**

**eMethods 4. Detailed Statistical Methods**

**eTable 1. DIC for model selection**

**eTable 2. Risk of bias of individual trials**

**eTable 3 Computing Method of Mean and Standard Deviation of Merged Groups**

**eFigure 1. Risk of bias summary**

**eFigure 2. Funnel plot**

**eFigure 3. Network plots**

**eFigure 4 Brooks-Gelman-Rubin diagnosis plot for each strategy**

**eFigure 5 Trace plot for each strategy**

**eMethods 1: Search strategy**

Data sources: PubMed, the Cochrane Library, EMBASE, EBSCO, Web of Science, and CINAHL databases from inception through Sep 30th, 2018.

Predefined key words and terms: “pulmonary embolism, pulmonary thromboembolism (PTE) [MeSH]) AND (fibrinolysis* OR thrombolytic* OR Thrombolytic therapy/methods* OR streptokinase OR urokinase OR Abbokinase OR tissue plasminogen activator OR tPA OR recombinant tissue-type plasminogen activator OR rt-PA OR alteplase OR prourokinase OR Umbralina OR tenecteplase) AND (randomized controlled trial [pt] OR controlled clinical trial [pt] OR randomized [tiab] OR placebo [tiab] OR clinical trials as topic [mesh: noexp] OR randomly [tiab] OR trial [ti]) NOT (animals [mh] NOT humans [mh])”.

**eMethods 2: Drug doses**

**Post-thrombolysis Anticoagulation Group:**

- Streptokinase group received 1500000 IU of SK over 1 hour by the peripheral vein, followed by a bolus of 10000 U of heparin and then a constant infusion of 1000 U/hr of heparin titrated to a partial thromboplastin time (PTT) of 2-2.5 times control. The heparin group followed the same regimen, but without streptokinase.
- Patients receiving urokinase were given a loading dose of 2,000 Thrombolytic Agents (CTA) units/lb of body weight followed by 2,000 CTA units/lb/hr, while the patients given sodium received a loading dose of 75 units of heparin/lb of body weight followed by 10 units/lb/hr. After termination of the 12-hour test infusion, all patients received heparin intravenously for a minimum of five days followed by orally administered therapy. In this postinfusion period heparin was administered intravenously either intermittently every four to six hours in doses sufficient to prolong the Lee-White clotting time to 30 to 45 minutes within one hour before the next dose, or, the latter level was maintained by a constant heparin infusion.
- Streptokinase was administered by constant peripheral vein infusion as a loading dose of 250,000 IU in 5% dextrose over a 20 to 30-minute period, followed by maintenance dosage of not less than 100,000 IU/hour for 18 to 72 hours. The rate of infusion of the maintenance dose was adjusted on the basis of the thrombin time at periodic intervals during treatment (0, 2, 4, 8, 12, 16, 20, and 24 hours, and at 12-hour intervals thereafter). The aim was to maintain the thrombin time at between two and five times its control value. If it exceeded five times the control value, the infusion rate was doubled every 4 hours until the thrombin time fell within the desired range. Conversely, if the thrombin time fell from the desired range to less than two times the control value, the rate was halved every 4 hours until the thrombin time was in the desired range. Fibrinogen, plasminogen, and fibrin/fibrinogen degradation product levels were determined along with the thrombin time, although the results were not used to adjust dose rates. After the termination of streptokinase therapy and when the thrombin time had reached less than twice the control value, patients were given heparin, 1200 =b 300 units/hour, by infusion. In patients treated with heparin alone, a loading dose of 1500 units of heparin per kilogram of body weight was given, followed by a similar constant rate infusion. Heparin infusions were monitored by Lee-White clotting times or activated clotting times (ACT) at 4-hour intervals during the first 24 hours, and subsequently at 12-hour intervals. A Lee-White clotting time of 25 to 35 minutes or an ACT of 145 to 160 seconds was considered indicative of proper heparin dosage. Long-term anticoagulation with warfarin sodium replaced heparin therapy when the protrombin time had reached two to two and one-half times the control and was maintained for 3 days.
- The dose of rt-PA was 100mg infused through a peripheral vein over 2 h (50 mg/h). Heparin was then administered at a concentration of 1000 units/h when the thrombin time or partial thromboplastin time (PTT) was less than twice control. Subsequently, continuous intravenous heparin was administered to achieve a target PTT of 1.5-2.5 times the upper limit of normal. For patients randomized to heparin alone, the initial dose was 5000 units as a bolus followed by 1000 units/h as a peripheral intravenous infusion. 4 h after randomization a PTT was obtained. Subsequent heparin doses were administered to achieve a target PTT of 1.5-2.5 times the upper limit of normal.

**Pre-thrombolysis Anticoagulation Group:**

- Patients who were assigned to undergo fibrinolysis received a single weight-based intravenous bolus (given over a period of 5 to 10 seconds), dose ranged from 30 mg to 50 mg, depending on body weight. The administration of unfractionated heparin was started as an intravenous bolus immediately after randomization in both groups; the bolus was not administered to patients who had already received an intravenous bolus or infusion of unfractionated heparin. The initial bolus of unfractionated heparin was also omitted in patients receiving a therapeutic dose of low-molecular-weight heparin or fondaparinux, and the start of the infusion was delayed until 12 hours after the last injection of low-molecular-weight heparin or until 24 hours after the last injection of fondaparinux. The heparin infusion rate was adjusted to achieve and maintain an activated partial-thromboplastin time that was 2.0 to 2.5 times the upper limit of the normal range, corresponding to therapeutic heparin levels (equivalent to factor Xa inhibition of 0.3 to 0.7 IU per milliliter).
- All patients received either unfractionated heparin or subcutaneous enoxaparin, with initial preference given to the latter drug. Enoxaparin was given to 48 of 61 (79%) of the patients in the thrombolysis group (TG) and 49 of 60 (81%) in the control group (CG). Administration of unfractionated heparin was determined by the presence of renal insufﬁciency or patient preference. In the TG, enoxaparin was given as 1 mg/kg subcutaneously twice daily, with the initial dose not to exceed 80 mg. For unfractionated heparin in the same group, it was given at 70 U/kg as a bolus but not to exceed 6,000 U, with subsequent dose adjustment to keep the activated partial thromboplastin time at 1.5 to 2 times the baseline value. Although tPA was infused, the maintenance dose of unfractionated heparin was kept at 10 U/kg/hour and not to exceed 1,000 U/hour. At 3 hours after termination of thrombolysis, the dose of unfractionated heparin was increased to 18 U/kg/hour. In the CG, enoxaparin was given at 1 mg/kg subcutaneously twice daily and unfractionated heparin at 80 U/kg as a bolus followed by 18 U/kg/hour, with the same partial thromboplastin time target. In the present study, tPA was the only thrombolytic drug used. The dose of tPA was ≤ 50% of the standard dose (100 mg) commonly used for the treatment of PE, which we termed “safe dose” thrombolysis. For patients weighing ≥50 kg, the total dose was 50 mg, which was given as a 10-mg bolus by an intravenous push within 1 minute followed by infusion of the remaining 40 mg within 2 hours. For those weighing <50 kg, the total dose was calculated as 0.5 mg/kg, which was given as a 10-mg initial bolus followed by the remainder within 2 hours. Warfarin was started at admission in all patients.
- When ﬁnal diagnosis of SPE was done, they received the assigned treatment: 100 mg of alteplase (Actilyse as a 10-mg bolus, followed by a 90-mg intravenous infusion over a period of 2 hours) or matching placebo. In addition to alteplase or placebo, both groups continued to receive unfractionated heparin treatment (1000 U/hr and/or accordingly activated partial thromboplastin time [aPTT]), in combination with warfarin (started on day 1 after randomization), until the international normalized ratio was within the therapeutic range for 2 consecutive days; after this point, heparin was stopped, and only warfarin was kept after discharge and during follow-up.
- Tenecteplase was given as an intravenous weight adjusted bolus (given over 5 seconds) at a dose ranging from 30 to 50 mg, with a 5mg step every 10 Kg from < 60 to ≥ 90 Kg. Bolus of Tenecteplase or placebo were scheduled to be given within 6 hours from baseline echocardiography. UH 80 IU/kg intravenous bolus, followed by an infusion of 18 IU/kg/h. Maximum bolus dose allowed was of 5000 IU (4000 IU for patients with body weight <67 kg). Heparin dose was adjusted in order to achieve and maintain the activated partial thromboplastin time (aPTT) at 2.0-2.5 x control. Vitamin K antagonists were started preferably on the same day of study treatment administration or as soon as possible. Heparin was continued until the INR was in the therapeutic range (2.0 to 3.0) in two consecutive days. For patients already on heparin treatment at the time of inclusion in the study, bolus administration was omitted.
- Patients believed to have acute sub-massive pulmonary embolism, as previously defined, received an intravenous bolus of 5000 U of unfractionated heparin before undergoing further diagnostic workup. Patients who met the inclusion criteria and were enrolled in the study were then randomly assigned to receive 100 mg of alteplase (Actilyse, Boehringer Ingelheim Pharma) as a 10-mg bolus, followed by a 90-mg intravenous infusion over a period of two hours. In addition to alteplase or placebo, patients in both groups received an intravenous infusion of unfractionated heparin. The infusion was started at a rate of 1000 U per hour, and the rate was subsequently adjusted to maintain the activated partial-thromboplastin time at 2.0 to 2.5 times the upper limit of normal. Overlapping oral anticoagulant therapy was started on day 3 after randomization, and the dosage was adjusted to maintain an international normalized ratio of 2.5 to 3.5.
- All patients received an initial intravenous heparin bolus of 5,000 units followed by heparin by continuous infusion at a starting dose of 30,000 units for the first 24 hours (20,000 units in 500 ml of 2/3:1/3 dextrose/saline solution infused at 31 ml per hour). The heparin dose was adjusted daily according to the results of laboratory monitoring using the activated partial thromboplastin time (AVTT) to maintain the results between 55 and 75 seconds (corresponding to approximately 1.5-2 times control using Dade actin-FS PTT reagent). Patients received rt-PA (0.6 mg ideal body weight reconstituted in 50 ml sterile water) by bolus injection over 2 minutes through aside port in the intravenous tubing. The heparin was interrupted only for the duration of the study drug infusion. The same procedure was followed for patients who received the saline solution placebo.
- During the diagnostic procedure all patients received heparin (10000 IU. as an intravenous bolus injection). Later. patients were randomly allocated to two groups, and given either 1) a 2-h infusion of alteplase (100 mg [10-mg bolus dose plus 50 mg in h1 and 40 mg in h2; Actilyse, Boehringer Italia S.p.A.] through a peripheral vein) followed by intravenous heparin; or 2) a heparin infusion. The suggested rate of heparin infusion was 1.750 IU/h for 7 to 10 days. However, the study protocol recommended adjustment of the rate of heparin infusion to a target range of activated partial thromboplastin time that was two to three times the mean normal value of each laboratory.
- Patients randomly assigned to received Enoxaparin (1 mg/kg subcutaneous twice a day) as an anticoagulant versus Enoxaparin (1 mg/kg subcutaneous twice a day) plus Alteplase (100 mg/90 min) or Streptokinase (1500000 u/2 hours) as an anticoagulant plus a thrombolytic. Overlapping oral anticoagulant therapy was started on day 3 after randomization, and the Warfarin dosage was adjusted to maintain an international normalized ratio of 2.5 to 3.5.
- Patients received rt-PA produced by Genentech, Inc (rt-PA, Genentech G11014). The rt-PA was administered intravenously at a rate of approximately 1 mg/min. Initially, a dose of 80 mg was selected as a modest dose on the basis of experience with myocardial infarction. After one major hemorrhage, the investigators reduced the dose to 40 mg in the interest of further reducing the risk of bleeding. Five patients received rt-PA 40 mg, one patient received rt-PA 64 mg (the result of hemorrhage interrupting an intended 80 mg treatment), and three patients received rt-PA 80 mg. The 40 mg dose of rt-PA was administered over 40 minutes. The 80 mg dose was administered over 90 minutes. During the infusion of rt-PA, heparin was administered to all but one patient in an open fashion; doses were determined by the attending physician. In one patient, heparin was discontinued during the double-blind administration of rt-PA. After informed consent, all patients were treated with full-dose low-molecular-weight heparin (LMWH), 1 mg/kg enoxaparin, or weight-based dalteparin, 200 units/kg, administered subcutaneously prior to injection of study drug or placebo. If the patient was receiving unfractionated heparin, this was discontinued and LMWH was started. Patients received tiered dose Tenecteplase in accordance with the TNKase insert (Genentech Inc., San Francisco, CA, USA). Quality of anticoagulation was assessed by the time in therapeutic range (TTR), deﬁned as the percentage of INR measurements that were found to be between 2 and 3 in the time-frame of 1 week after discharge and 90-day follow-up.
- Streptokinase (Streptase, Behringwerke, Marburg-Lahn, West Germany), supplied by Norske Hoechst, was given in standard doses. After a loading dose of 250000 IU dissolved in 20 mlO.9% NaCl given i.v. in 20 min, a maintenance dose of 100000 IU/hour was given by continuous i.v. infusion (Infusomat, Braun, Meslingen, West Germany). The infusion was continued until control pulmonary angiography after 72 hours. Streptokinase was then discontinued and oral anticoagulation with warfarin was started. In addition, when the thrombin clotting time was less than twice the normal control value, heparin was given i.v. in doses of 10000-30000 IU/day until therapeutic values of Thrombotest were obtained. To avoid anaphylactic reactions, 100 mg of soluble hydrocortisone were given i.v. before the loading dose of streptokinase, whereafter prednisone, 10 mg 3 times daily, was given until discontinuation of streptokinase. Heparin (Apotekernes Laboratorium, Oslo, Norway) was given in an initial dose of 15000 IU i.v., followed by a maintenance dose of 30000 IU/day as a continuous i.v. infusion. The dose of heparin was subsequently adjusted according to the thrombin clotting time. (final concentration of 10 NIH U/ml of thrombin) to maintain an in vivo heparin effect corresponding to a heparin concentration of 0.5-1.0 IU/ml in normal plasma. The daily dose thus varied from 30000 to 60000 IU. Warfarin was started after control angiography and heparin was discontinued when therapeutic values of Thrombotest were obtained. As a mean, heparin was given for 7 days, but was continued for 17 days in one patient with symptoms of persistent massive embolism.

**eMethods 3. Event definitions**

The definitions of event definitions were consistent with the prespecified definition in the primary research.

**eMethods 4: Detailed statistical methods**

Bayesian network meta-analysis

All analyses were performed using R i386 (version 3.2.2, 3 chains were used, including 50000 burn-in iterations followed by 100000 iterations), and JAGS (version 4.0.0).

Deviance information criterion was adopted to determine the model-used (fixed vs. random-effects) for individual outcomes using the approach in Kew et al. For each outcome, the model with the lowest DIC value was used. Using this approach, fixed-effects model was used for primary and secondary outcomes, and random-effects model was used for safety outcomes. The DIC values are listed in the eTable 1 of the Supplement.

For each outcome, a network was generated incorporating all studies that reported at least one event in at least one study group. Studies in which no events occurred were excluded from the network, as these studies do not contribute to the treatment effect estimate.

The Markov Chain Monte Carlo (MCMC) method was used and convergence was assessed using the Brooks-Gelman-Rubin, using a cut-off of 1.05. 95% Crl that do not cross 1 were taken to represent statistical significance. I^2^ was used to measure heterogeneity[^9^](#_ENREF_9) (I^2^ < 25% was considered mild, I^2^ < 75% was moderate and I^2^ > 75% was severe[^10^](#_ENREF_10)).

**eTable 1: Deviance information criterion for model selection (fixed- vs. random-effects)**

| Outcome | Model | Deviance Information  Criterion (DIC) | Accepted Model |
| --- | --- | --- | --- |
| Major bleeding | Random | 38.754 | Random |
|  | Fixed | 42.479 |  |
| Recurrence | Random | 39.908 | Random |
|  | Fixed | 40.122 |  |
| All-cause mortality | Random | 55.210 | Random |
|  | Fixed | 61.404 |  |
| Composite outcome | Random | 54.385 | Random |
|  | Fixed | 82.899 |  |

**eTable 2: Risk of bias of individual trials**

| Source | Sequence generation | Allocation concealment | Blinding | Detection Bias | Attrition |
| --- | --- | --- | --- | --- | --- |
| B. Ly, H., et al. 1978 | Low | Low | Low | Low | Low |
| A Cooperative Study. 1970 | Low | Unclear | Low | Low | Low |
| Goldhaber, S. Z., et al.1993 | Low | Low | High | Low | Low |
| Charles T., et al.1979 | Low | Unclear | Unclear | Low | Low |
| Jerjes- S, C., et al. 1995 | Low | Low | Unclear | Low | Low |
| Konstantinides, S., et al.2002 | Low | Low | Low | Low | Low |
| Meyer, G., et al. 2014 | Low | Low | Low | Low | Low |
| Sharifi, M., et al. 2013 | Low | Unclear | Unclear | Low | Low |
| Fasullo, S., et al. 2011 | Low | Low | Low | Low | Low |
| Becattini, C., et al. 2010 | Low | Low | Low | Low | Low |
| Levine, M., et al. 1990 | Low | Unclear | Low | Low | Low |
| Dalla-Volta, S., et al. 1992 | Low | Low | High | Low | Low |
| Taherkhani, M., et al.2014 | Low | Low | Low | Low | Low |
| PIOPED Investigators 1990 | Low | High | Low | Low | Low |
| Kline, J. A., et al. 2014 | Low | Low | Low | Low | Low |

**eTable 3 Computing Method of Mean and Standard Deviation of Merged Groups**

| Items | Group 1 | Group 2 | Merged Group |
| --- | --- | --- | --- |
| Sample size | N_1_ | N_2_ | N_1_+N_2_ |
| Mean | M_1_ | M_2_ | (N_1_M_1_+ N_2_M_2_)/(N_1_+N_2_) |
| Standard Deviation | SD_1_ | SD_2_ | SQRT(((N_1_-1) SD_1_^2^+(N_2_-1) SD_2_^2^+( N_1_N_2_) (M_1_-M_2_)^2^/(N_1_+N_2_))/(N_1_+N_2_-1)) |

**eFigure 1: Risk of bias summary**


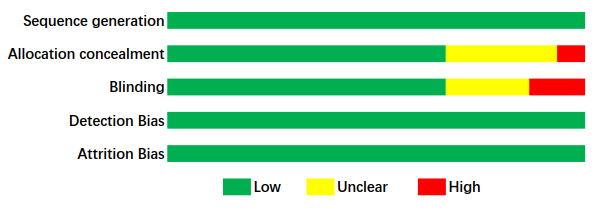


**eFigure 2: Funnel plot**





Funnel plot in the primary outcome of all-cause mortality (15 trials).

**eFigure 3: Network plots**

**Major bleeding events**


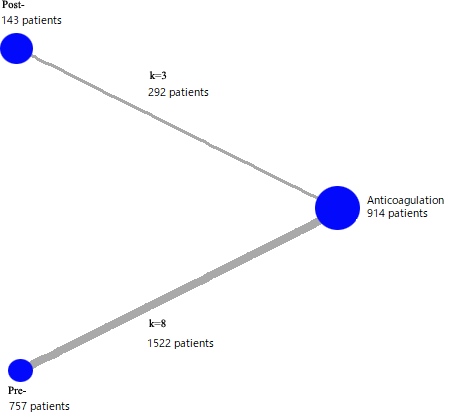


**Recurrence**


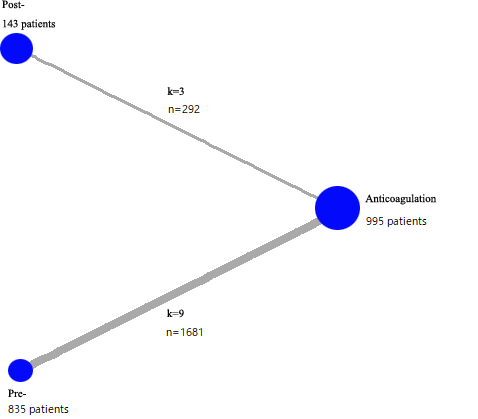


**All-cause mortality**


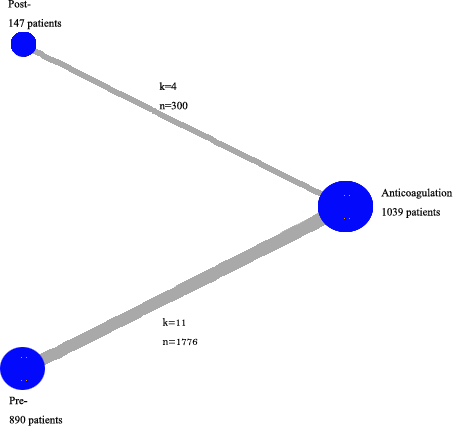


**Composed events**


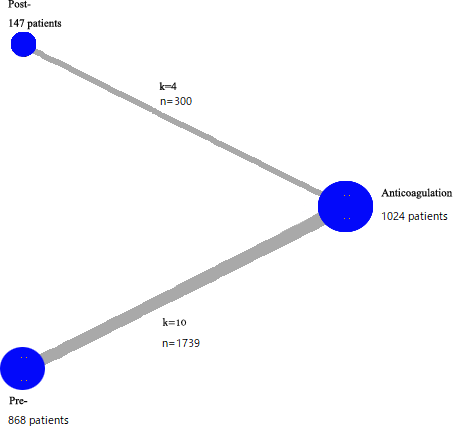


Graphical representation of networks for primary, secondary and safety endpoints. Connecting lines represent head-to-head comparisons (thickness proportional to number of trials) between drugs, indicated by nodes (size proportional to number of patients).

k - number of trials per comparison; n – number of patients per comparison.

**eFigure 4** **Brooks-Gelman-Rubin diagnosis plot for each strategy**


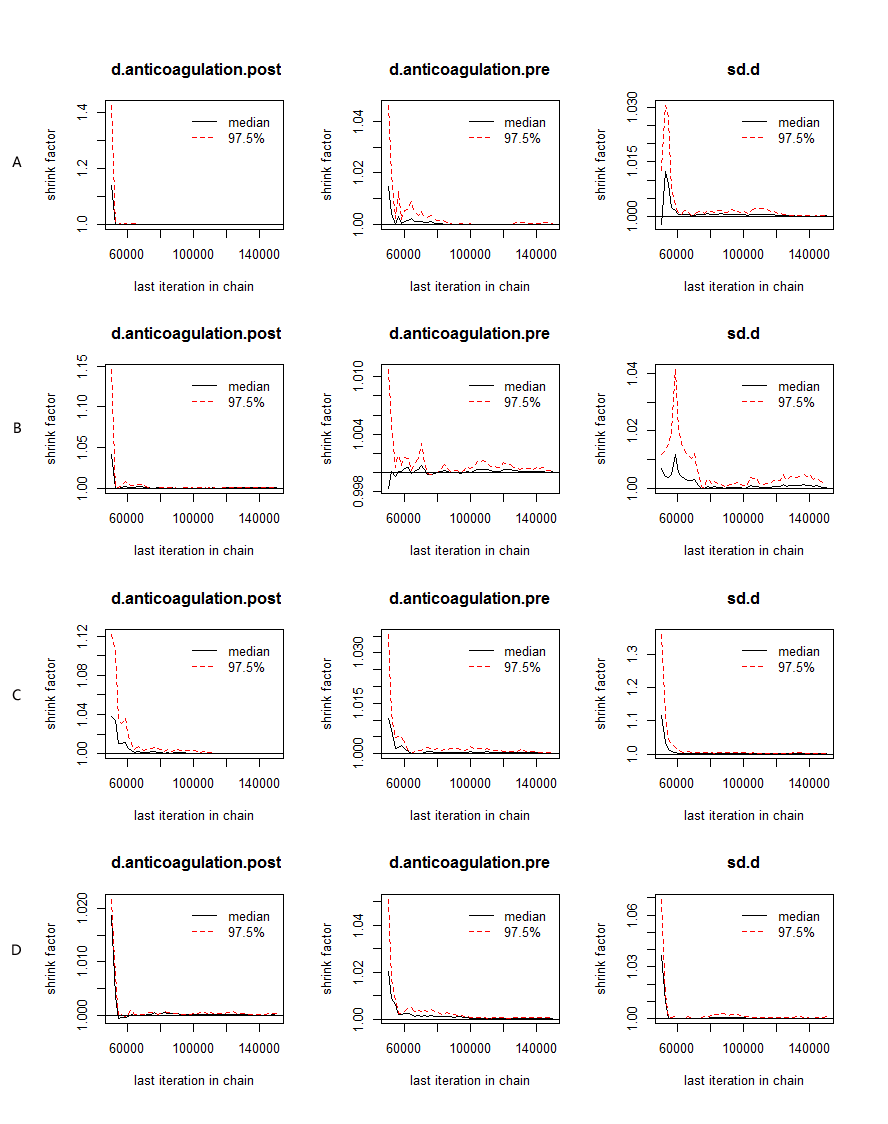


Note: A: Brooks-Gelman-Rubin diagnosis plot on major bleeding events. B: Brooks-Gelman-Rubin diagnosis plot on recurrent PE. C: Brooks-Gelman-Rubin diagnosis plot on all-cause mortality. D: Brooks-Gelman-Rubin diagnosis plot on composite outcome.

**eFigure 5 Trace plot for each strategy**


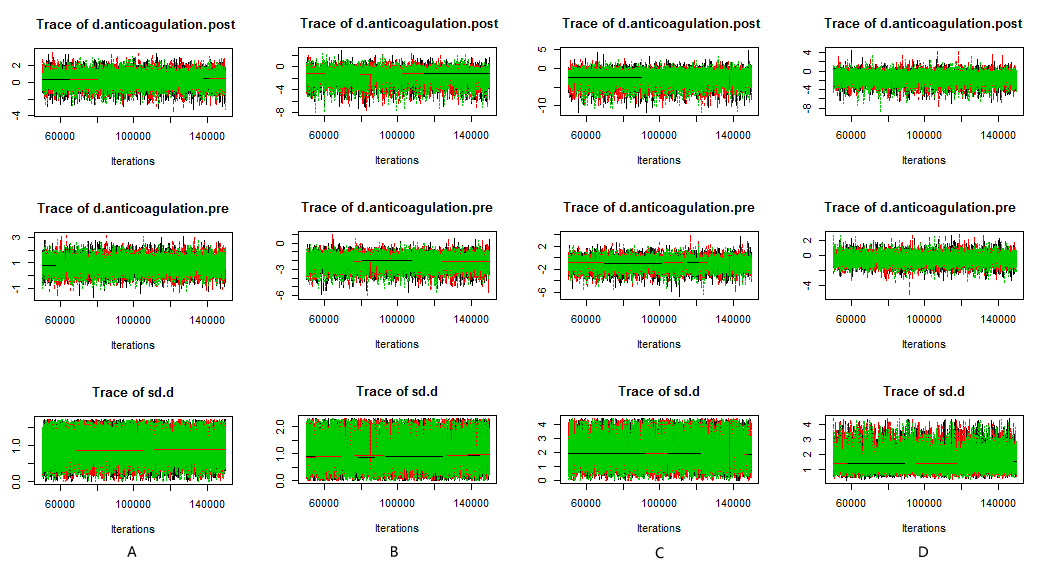


Note: A: Trace plot on major bleeding events. B: Trace plot on recurrent PE. C: Trace plot on all-cause mortality. D: Trace plot on composite outcome.
